# Synthesis of Calcium Phosphate by Microwave Hydrothermal Method: Physicochemical and Morphological Characterization

**DOI:** 10.1155/2024/2167066

**Published:** 2024-11-06

**Authors:** Ana Elisa Vilicev Italiano, Ricardo Luis Tranquilin, Danny Omar Mendoza Marin, Márcio Luiz dos Santos, Luís Geraldo Vaz

**Affiliations:** ^1^Department of Dental Materials and Prosthodontics, Araraquara School of Dentistry, Sao Paulo State University (UNESP), São Paulo, São Paulo, Brazil; ^2^Department of Biotechnology and Pharmacy, Sao Paulo Anhanguera University (UNIAN), São Paulo, São Paulo, Brazil; ^3^Department of Dentistry, Santa Catarina Federal University (UFSC), Florianópolis, Brazil

**Keywords:** biomaterials, bone grafts, calcium phosphates, hydrothermal synthesis, microwave-assisted

## Abstract

Bone loss in the alveolar ridge is a factor widely studied by dentists in implant surgeries, as it poses a major challenge for aesthetic and functional recovery in patients with large maxillary bone defects. Synthetic biomaterials function as grafts designed to replace and remodel bone tissue. Calcium phosphate is a biomaterial that has good properties such as biocompatibility and bioactivity, making it a reference in bone replacement treatments. A synthetic biomaterial such as calcium phosphate can be obtained by various synthesis techniques. The microwave hydrothermal method (HTMO) is a pathway that allows changes in synthesis parameters and significantly increases the transmission efficiency of materials such as synthetic calcium phosphate derivatives. The study proposes obtaining a biomaterial for bone grafting based on calcium phosphate by the microwave HTMO and evaluating its microstructural and physicochemical characteristics. The parameters tested in this process were temperature and reaction time. The calcium phosphate particulates were obtained by the microwave HTMO at temperatures of 110°C and 130°C for 60 min and calcined at 300°C, 500°C, and 700°C. Microstructural and physicochemical characterization analyses were carried out using scanning electron microscopy, Fourier transform infrared, and X-ray diffraction. The results obtained showed the presence of more than one calcium phosphate biological interest phase, as hydroxyapatite (HA), tricalcium phosphate (*β*-TCP), and octacalcium phosphate (OCP), highlighting with increasing calcination temperature, the *β*-TCP phase becomes evident. The proposed synthesis method was efficient in obtaining a biomaterial with suitable physical and chemical characteristics, with an association of crystalline phases of biological interest related to the increase in synthesis temperature and calcination temperature.

## 1. Introduction

Bones, as support matrices for the body, can present different types of integration between organic and inorganic material, which produces considerable variations in mechanical properties. The ratio between the two components reflects the relationship between hardness (high inorganic content) and elasticity or resistance to fracture (low inorganic content). Besides performing these functions, bones also serve as a storage site for calcium, phosphate, and other ions, regulating their release and absorption in a controlled manner to uphold a stable concentration of these ions in bodily fluids such as interstitial fluid and blood [1–4]. The lack of bone in the alveolar ridge has been a major problem in the aesthetic-functional recovery of patients who have suffered dentoalveolar trauma, traumatic tooth extractions, congenital absence of teeth, pathologies involving the maxilla and mandible, as well as infections. Bone loss can occur due to periodontal disease, traumatic surgery, or physiological reasons due to lack of ridge function or inadequate prosthetic load [1, 2].

Much is being studied about the technological development of biomaterials to selectively influence the tissue response of the receiving bed, as bioceramics, which should lead to new bone formation by controlling the quality and quantity of bone within the receiving area [1, 2]. Synthetic biomaterials function as grafts designed to replace and remodel various types of human tissue. These synthetic grafts function as carriers of cells and various molecules that act as adjuvants in tissue repair [1, 3, 5]. Calcium phosphate derivatives are known for their biocompatibility, lack of immunogenic reactions and apparent ability to bind to host tissue. These benefits can be explained by the chemical nature of these materials, which are basically made up of calcium and phosphate ions, actively participating in the ionic balance between the biological fluid and the ceramic [2, 6, 7]. Calcium phosphate has good properties as a biomaterial, biocompatibility, and bioactivity, as well as appreciable mechanical properties, which has made it a reference in bone replacement treatments. The characteristics associated with hydrophilicity, which allows the surface to be moistened by body fluids, favor the proliferation of bone cells (osteoblasts) [7–10]. The characteristics of calcium phosphate derivatives depend on a series of factors relating to the synthesis process, such as impurities, precursor reagents, crystal morphology and size, concentration, pH, and temperature. In addition, the bioactivity of these materials also depends on the heat treatment profile for drying and sintering the material [9, 11].

Santos et al. [12] highlight the importance of the hydroxyapatite (HA) phase in bone repair, since it presents structural and chemical similarities with the bone mineral section, in addition to presenting properties such as biocompatibility, bioactivity, and osteoconductivity.

Some methods for preparing calcium phosphate synthetically can be found to literature, such as precipitation, sol-gel process, spray pyrolysis, hydrothermal synthesis, emulsion processing, and mechanochemical method. Some authors have demonstrated the use of microwave irradiation in organic chemical transformations, considering short reaction times, increased product yield, greater product purity and reduced side reactions due to microwave heating [13, 14]. The microwave heating synthesis method is used in chemical processes, especially for organic and inorganic products. In general, microwave energy goes beyond the external surface of the system, reaching its interior, allowing the reaction to occur homogeneously, reducing reaction time, in addition to not requiring postreaction cooling processes [13–16].

Leonelli and Komarneni [17] broadly developed the microwave hydrothermal process for the synthesis of inorganic materials on nanometric scale and studies demonstrated advantages such as rapid heating to the reaction temperature, increased reaction kinetics, elimination of metastable phases, and formation of new phases.

The objective of this study was to investigate the synthesis of calcium phosphate utilizing the microwave hydrothermal method (HTMO) at temperatures of 110°C and 130°C for a 60 min duration, followed by calcination at 300°C, 500°C, and 700°C, to assess the effects of time and temperature. The resulting materials were subjected to characterization using scanning electron microscopy (SEM), Fourier transform infrared (FTIR) spectroscopy, and X-ray diffraction (XRD).

## 2. Materials and Methods

### 2.1. Synthesis of Materials

The synthesis via the microwave HTMO involved dissolving Ca (OH)_2_ at a concentration of 1 mol/L in distilled and deionized H_2_O. Phosphoric acid (H_3_PO_4_) was then added to the solution in a round-bottomed flask, which was heated and continuously stirred at 90°C. Subsequently, the suspension was transferred to a Teflon reactor and subjected to the microwave hydrothermal process (HMO-100, LIEC) at temperatures of 110°C and 130°C for 60 min. After centrifugation, the resulting powders were dried in a vacuum oven and subjected to heat treatment at temperatures of 300°C, 500°C, and 700°C for 1 h. The samples were divided into 8 groups, as outlined in Table 1.

### 2.2. Physical and Chemical Characterization

The used techniques were FTIR (Bruker Equinox 55), with an accessory for diffuse reflectance in the range of 400–4000 cm^−1^, XRD on a Rigaku PC-Max 2500 diffractometer with an angular scan between 10° and 60°, using CuK*α* radiation (*λ* = 1.5418 Å), voltage of 30 kV and current of 15 mA, with a speed of 0.02 degrees every 40 s and SEM Carl Zeiss, model supra 35-VP.

## 3. Results and Discussion

**Figure 1:**
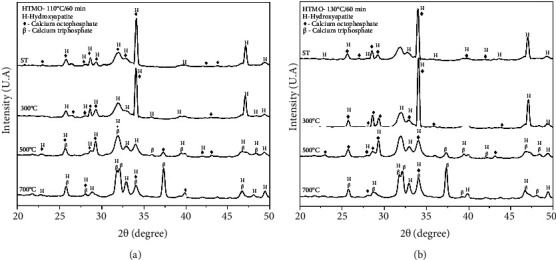
(a) X-ray diffractograms of the samples obtained by synthesizing calcium phosphate for 60 min at 110°C using the microwave hydrothermal method (HTMO). The curves represent the samples without heat treatment, and calcined at 300°C, 500°C, and 700°C. HA (hydroxyapatite), OCP (octacalcium phosphate), and TCP (tricalcium phosphate). (b) X-ray diffractograms of the samples obtained by synthesizing calcium phosphate for 60 min at 130°C using the microwave hydrothermal method (HTMO). The curves represent the samples without heat treatment and calcined at 300°C, 500°C, and 700°C. HA (hydroxyapatite), OCP (octacalcium phosphate), and TCP (tricalcium phosphate).

Figure 1(a) shows the diffractograms obtained by XRD of the samples synthesized at 110°C for 60 min using the microwave HTMO and calcined at 300°C, 500°C, and 700°C, which belong to groups G1, G2, G3, and G4.

Figure 1(b) shows the diffractograms obtained by XRD of the samples synthesized at 130°C for 60 min using the microwave HTMO and calcined at 300°C, 500°C, and 700°C, which belong to groups G5, G6, G7, and G8.

XRD analysis showed that all synthesis temperatures, without or with heat treatment, led to the formation of HA, as well as the presence of the OCP phase, which is recognized as a precursor to the HA phase. It should also be noted that the characteristic peaks for HA show greater intensity and better resolution. This could mean that the material obtained is more crystalline. The 2*θ* values located within the 20° and 50° range correspond to the main peaks of the calcium phosphate phases of biological interest: HA, *β*-TCP, and OCP. The results found in this study can be confirmed with the work described by Lak et al. [18] and Méndez-Lozano et al. [19], who in their investigations on samples of calcium phosphate derivatives analyzed by XRD, identified HA as the main crystalline phase, evaluating it as an indication of the material's purity. Lak et al. [18] states the existence of sharp peaks and high intensity in the analysis of diffractograms confirming the formation of crystalline HA after microwave irradiation, showing differentiated XRD patterns compared to the standard file and other works that showed HA nanostructure using microwave irradiation at longer times [18–20].

This study corroborates the results presented to Santos et al. [21] among other authors, in which they demonstrate in physicochemical analyzes the formation of multiphase calcium phosphate according to the calcination temperature to which the material was exposed at temperatures above 300°C they observed the phases of amorphous phosphate (ACP 1 and 2), HA_d_ (calcium-deficient HA phase) and HA, while at temperatures above 600°C they observed the phases HA_d_, HA, and *β*-TCP, as shown in the following reactions [12, 21, 22].

(1) $$\begin{array}{c} \text{ACP}1⟶\text{ACP}2⟶{\text{HA}}_{d}⟶\text{HA}\\ {\text{Ca}}_{10}{\text{⁣}\left({\text{PO}}_{4}\;{\text{CO}}_{3}\right)}_{6}{\text{⁣}\left(\text{OH}\right)}_{2}\underset{⟶}{\geq 600^{{}^{\circ}}C}\beta -{\text{Ca}}_{3}{\left({\text{PO}}_{4}\right)}_{2\text{⁣}}+{\text{Ca}}_{10}{\left({\text{PO}}_{4}\right)}_{6}{\left(\text{OH}\right)}_{2}+{\text{CO}}_{2}↑ \end{array}$$

In the diffractograms Figures 1(a) and 1(b), the OCP phase is represented by the reflection peaks that correspond to the reflection planes that corroborate JCPDS datasheet no. 79.423. In the samples treated at 500°C and 700°C, it is evident that the OCP phase is less pronounced and exhibits lower crystallinity, while the HA and *β*-TCP phases are more prominent. This is indicated by peaks corresponding to specific planes, such as 200, 002, 102, 210, 211, 300, 202, 212, 130, 131, 222, 312, and 213 for HA, according to JCPDS technical file no. 731731, and peaks corresponding to planes 024, 1010, 0210, 128, 2110, 1016, 4010, 146, 1211, 404, 2116, 214, 220, and 238 for TCP, as per JCPDS technical file no. 86.1585. These results are consistent with previous studies where researchers correlated the observed peaks in diffractograms with crystallite sizes. It has been observed in the synthesis of calcium phosphate using microwave irradiation that longer synthesis times lead to an increase in crystallite size. According to the authors Natalia L [11], Lack A [18], and Méndez-Lozano [19], this result was expected since longer syntheses provide the crystal with more time for nucleation and growth and present in them analyzes a 13% increase in the size of the crystallite when compared to the methods of conventional synthesis [11, 18, 19].

The infrared analyses showed results that corroborate the XRD analyses, both for the samples without heat treatment (ST) (G1 and G5) and for the samples calcined at 300°C (G2 and G6), 500°C (G3 and G7), and 700°C (G4 and G8).

**Figure 2:**
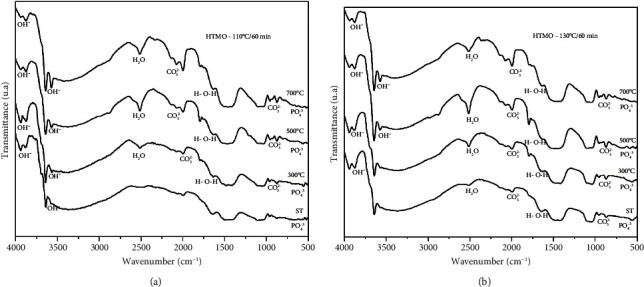
(a) Infrared spectra of the samples obtained by synthesizing calcium phosphate for 60 min at 110°C using the microwave hydrothermal method (HTMO). The curves represent the samples without heat treatment (ST) and calcined at 300°C, 500°C, and 700°C. (b) Infrared spectrum of the samples obtained by the synthesis of calcium phosphate conducted for 60 min at 130°C using the microwave hydrothermal method (HTMO). The curves represent the samples without heat treatment (ST) and calcined at 300°C, 500°C, and 700°C.

The infrared spectra obtained for the samples synthesized by the microwave HTMO at temperatures of 110°C (a) and 130°C (b) for 60 min are depicted in Figures 2(a) and 2(b), respectively. According to the FTIR analysis, the appearance of bands at 3939 cm^−1^, 3873 cm^−1^, 3783 cm^−1^, 3691 cm^−1^, 3640 cm^−1^, and 3572 cm^−1^ signifies axial stretching deformations of phosphate groups, associated with the OH group. Additionally, bands at 523 cm^−1^ and 694 cm^−1^ in the samples treated at 700°C indicate the presence of phosphate ions (PO_4_^3−^), reflecting angular deformations of this group. Moreover, bands observed at 3640 cm^−1^, 1645 cm^−1^, and 1647 cm^−1^ in the samples treated from 300°C can be attributed to H-O-H deformation.

The curves indicate reduced intensity in the bands 3691 cm^−1^, 2142 cm^−1^, 1793 cm^−1^, 1749 cm^−1^, 957 cm^−1^, 874 cm^−1^, and 792 cm^−1^. A broad band was observed between 2700 cm^−1^ and 3800 cm^−1^, with maximum intensity around 3640 cm^−1^, which would reflect the combination of water and powder Ca_3_(PO_4_). The greater solubility of the ACP phase in relation to HA becomes an important characteristic for its use as a biomaterial, as it provides a higher rate of degradation in the biological environment. A soluble material allows the exchange of Ca^2+^ and PO_4_^−3^ ions with the biological environment, facilitating bone growth [19, 23].

The bands observed at 2513 cm^−1^ and 2347 cm^−1^ are indicative of the presence of water, while bands at 2142 cm^−1^, 2077 cm^−1^, 957 cm^−1^, 929 cm^−1^, 874 cm^−1^, and 792 cm^−1^ are associated with carbonate (CO_3_^2−^) presence. Additionally, the band at 1753 cm^−1^ suggests the presence of carbonates, linked to Type B substitution commonly found in biologically relevant calcium phosphates [18, 21, 23]. The absorption of these bands is sensitive to the type of substitution of the CO_3_^2−^ group, which can be associated with CO_2_ from the atmosphere during the dissolution, agitation, reaction, and calcination processes, or to the formation of carbonated HA due to the possibility of substitutions occurring in the PO_4_^3−^ ions or hydroxyl arising from HA to the CO_3_^2−^ ion [12, 21, 22], as follows:

.(2) $$\begin{array}{c} 2{\text{OH}}^{-}\left(\text{hidroxyapatite}\right)+{\text{CO}}_{2}\left(\text{air}\right)⟶{\text{CO}}_{3}^{2-}\left(\text{carbonated hidroxyapatite}\right)+H_{2}O \end{array}$$

The absorption of these bands is sensitive to the type of CO group substitution. In Figures 2(a) and 2(b) spectra, the functional groups corresponding to significant bands of calcium phosphate derivatives of biological interest are illustrated, as shown in Table 2.

Méndez-Lozano et al. [19] and Gubicza [24] discussed the presence of an extensive band between 4000 cm^−1^ and 3700 cm^−1^ in an analysis of the infrared spectrum curves, corroborating the data obtained from the XRD, analyzed at the temperatures and time of synthesis, which revealed points of low crystallinity, characteristic of a biphasic material. According to the authors, the intensity of this peak is also related to the crystallinity of the powder, relating the HA phase, and even stating that the amorphous phase of the powder is more hydrophilic than its crystalline analog [19, 24].

**Figure 3:**
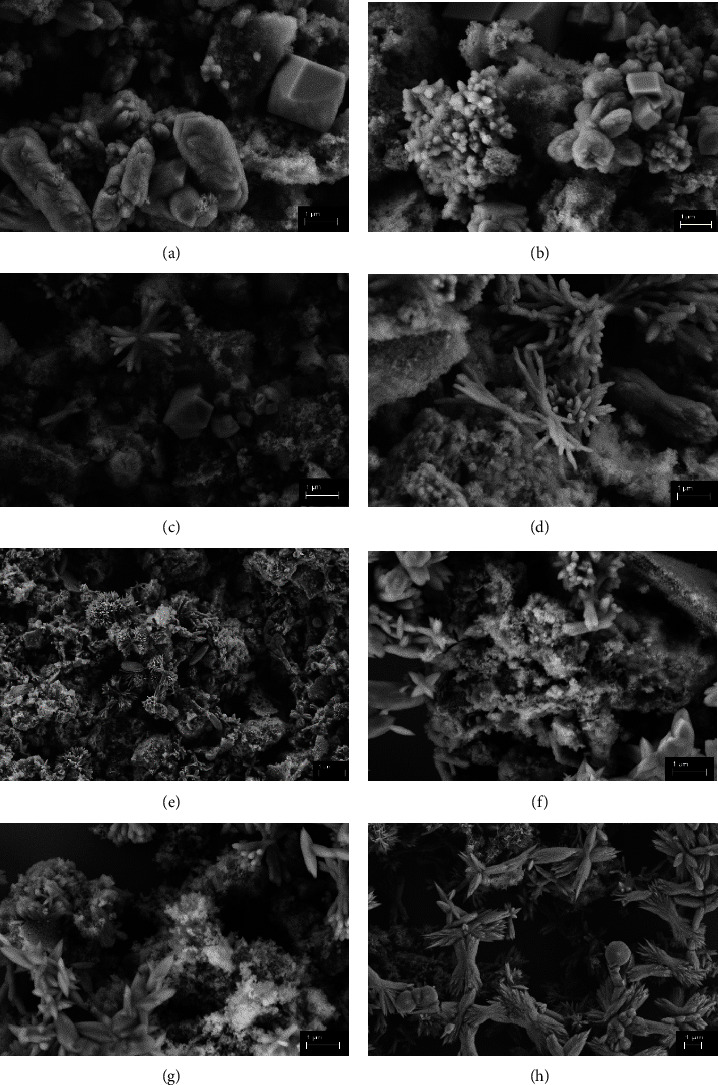
SEM micrographs of samples synthesized at 110°C (a) without heat treatment and calcined at 300°C (b), 500°C (c), 700°C (d), and 130°C (e) without heat treatment and calcined at 300°C (f), 500°C (g), and 700°C (h).

When comparing the results obtained by SEM analysis with the results obtained by XRD, it can be seen the materials characterized by this technique have more than one calcium phosphate phase which are of biological interest in bone regeneration, HA, *β*-TCP, and OCP. By analyzing Figures 3(a), 3(b), 3(c), and 3(d), it was possible to see that in the samples synthesized at 110°C for 60 min there is a predominance of agglomerates of particles tending towards a cubic shape, characteristic of less crystalline patterns, corroborating with the XRD, which indicates a more amorphous pattern [19]. However, in the samples calcined at 500°C (c) and 700°C (d), the formation of rods is observed, demonstrating an increase in the crystallinity of the material. Méndez-Lozano [19], among other authors, reports that the associated reaction time and temperature are factors that directly influence the morphology and growth direction of the HA crystal [25].

The samples synthesized at 130°C, shown in Figures 3(e), 3(f), 3(g), and 3(h), show the formation of apatite with flower-shaped agglomerates (< 5 *μ*m), which suggests a high surface area. It is recognized that the specific surface area is dependent on the synthesis temperature, the higher the temperature, the higher the surface energy, favoring the development of the flower shape of the HA [24, 25]. In an analysis of the micrographs taken, according to the mechanism suggested by Ma [26], the nucleation of calcium ions and self-organized growth occur under hydrothermal conditions, thus forming HA with a flower-like morphology [26]. The influence of microwave radiation on the hydrothermal process accelerates the crystallization of HA and helps to obtain it in a shorter synthesis time, with a high specific surface area, as well as providing a high yield. However, the high specific surface area and average particle size depend on the power (temperature) used in the microwave and the synthesis time [26, 27]. According to Lopes, Oliveira, and Esteves [28], the morphology of calcium phosphate particles can be spheroid and needle-shaped, as observed in samples synthesized at 130°C and calcined at 500°C and 700°C. The authors also reported that HA crystals tend to form agglomerates and porous shape by varying the size of the crystallites by increasing the calcination temperature, which suggests that the samples calcined at 700°C present dispersed particles with irregular sizes Figure 3(h) [28].

However, pressure can lead to an amorphous or partially disordered phase arrangement leading to larger crystalline sizes [11, 24, 29]. These considerations justify the fact that microwave-assisted hydrothermal systems produce larger crystals [29].

The biomaterial under study showed characteristics of multiphase bioceramics, corroborating the studies by Rustom, Poellmann, and Johnson [30], who also found similar results when evaluating a biphasic calcium phosphate HA/*β*-TCP. They suggest that the surface of the biphasic phosphate allows osteoclasts to act in a more natural way than pure HA or *β*-TCP, as well as being able to bind to living bone tissue and undergo natural actions during the bone remodeling process. They point out that biphasic calcium phosphate bioceramics are promising for traumatological applications in the repair of traumatized bone tissue and for the controlled release of drugs in bone structure treatments [25, 30, 31].

At times academic and industrial researchers are challenged to seek methods that do not release waste into the environment, considered by “green chemistry principles.” As microwave irradiation is recognized as an effective and nonionizing electromagnetic energy source, it attracts attention as an environmentally friendly and reliable method for multiphase processes. Microwave energy presents considerable efficiency in various processes that require selective heating [32]. When microwave heating is applied under isothermal conditions, thermally unstable elements absorb the energy generated. The thermal distribution is balanced by the activation energy of the microwaves, which is inversely proportional to the energy of the Ca and P ions, resulting in dissociation of the CaP compound phases as the temperature increases [33].

## 4. Conclusion

The primary inorganic constituent of bone tissue consists of calcium phosphate derivatives interwoven within a collagen matrix. A key advantage of calcium phosphates in bone regeneration biomaterials lies in their ability to modulate biodegradability and bioactivity by combining different calcium phosphate phases. In this investigation, calcium phosphate derivatives with biological relevance were synthesized using the microwave heating method. The study explored the effects of microwave irradiation time and energy on particle formation. Our findings indicate the microwave HTMO yielded satisfactory results for producing biologically relevant calcium phosphate derivatives, underscoring its potential as a bone regeneration material. Compared to conventional techniques, microwave HTMO offers advantages such as volumetric heating, ensuring homogeneous nucleation, and rapid saturation through dissolving precipitates. Analysis via XRD, FTIR, and SEM revealed characteristics of a biphasic phosphate capable of forming strong bonds with bone tissue, facilitating bone neoformation, a critical trait of bioactive materials. Structural assessments demonstrated the purity and crystallinity of the microstructures of the calcium phosphate phases, displaying distinct and well-aligned microstructures, as confirmed by morphological analysis. This study presented results in which synthesis time/temperature by HTMO was evaluated. The results presented in the synthesis at 130°C/1 h suggest that high sintering temperatures are not required to obtain the phases of biological interest from calcium phosphate. As preliminary experiments, the material obtained at 130°C without heat treatment presented (OCP) precursor phases of HA, and when calcined at temperatures of 500°C and 700°C, phases of biological interest of calcium phosphate (HA, *β*-TCP) were identified, which are important characteristics from the point of view of materials science for a biomaterial indicated for bone regeneration.

## Figures and Tables

**Table 1:** Groups corresponding to the synthesis and heat treatment conditions of the samples analyzed.

Groups	Synthesis temperature/weather	Calcination
G1	110°C/60 min	Without calcination
G2	110°C/60 min	300°C
G3	110°C/60 min	500°C
G4	110°C/60 min	700°C
G5	130°C/60 min	Without calcination
G6	130°C/60 min	300°C
G7	130°C/60 min	500°C
G8	130°C/60 min	700°C

**Table 2:** Functional groups corresponding to important bands in the FTIR spectrum of calcium phosphate derivatives.

Wavenumber (cm^−1^)	Groups (samples)	Functional groups
523–694	G4 e G8	PO_4_^3−^ (deformation)
871	G5 a G8	CO_3_^2−^ (stretch)
874	G1 a G4	CO_3_^2−^ (stretch)
2513	G1 a G8	H-O-H (stretch)
1647–3640	G2 e G6	H_2_O (deformation)
3572–3939	G1 a G8	OH^−^ (stretch)

## Data Availability

The authors declare that data supporting the conclusions of this study are available from the corresponding author upon reasonable request, reaffirming the information mentioned in the system and has already been readapted in the manuscript.
